# Combining Z-Score and Maternal Copy Number Variation Analysis Increases the Positive Rate and Accuracy in Non-Invasive Prenatal Testing

**DOI:** 10.3389/fgene.2022.887176

**Published:** 2022-06-02

**Authors:** Liheng Chen, Lihong Wang, Zhipeng Hu, Yilun Tao, Wenxia Song, Yu An, Xiaoze Li

**Affiliations:** ^1^ Department of Medical Genetics, Changzhi Maternal and Child Health Care Hospital, Changzhi, China; ^2^ School of Life Sciences, Fudan University, Shanghai, China; ^3^ Department of Pediatrics, Changzhi Maternal and Child Health Care Hospital, Changzhi, China; ^4^ Obstetrics Department, Changzhi Maternal and Child Health Care Hospital, Changzhi, China; ^5^ Human Phenome Institute, Zhangjiang Fudan International Innovation Center, MOE Key Laboratory of Contemporary Anthropology, Fudan University, Shanghai, China

**Keywords:** non-invasive prenatal testing (NIPT), copy number variations (CNVs), aneuploidies, prenatal diagnosis, birth defects, positive predictive value (PPV), z-scores

## Abstract

**Objective:** To evaluate positive rate and accuracy of non-invasive prenatal testing (NIPT) combining Z-score and maternal copy number variation (CNV) analysis. To assess the relationship between Z-score and positive predictive value (PPV).

**Methods:** This prospective study included 61525 pregnancies to determine the correlation between Z-scores and PPV in NIPT, and 3184 pregnancies to perform maternal CNVs analysis. Positive results of NIPT were verified by prenatal diagnosis and/or following-up after birth. Z-score grouping, logistic regression analysis, receiver operating characteristic (ROC) curves, and S-curve trends were applied to correlation analysis of Z-scores and PPV. The maternal CNVs were classified according to the technical standard for the interpretation of ACMG. Through genetic counseling, fetal and maternal phenotypes and family histories were collected.

**Results:** Of the 3184 pregnant women, 22 pregnancies were positive for outlier Z-scores, suggesting fetal aneuploidy. 12 out of 22 pregnancies were true positive (PPV = 54.5%). 17 pregnancies were found maternal pathogenic or likely pathogenic CNVs (> 0.5 Mb) through maternal CNV analysis. Prenatal diagnosis revealed that 7 out of 11 fetuses carried the same CNVs as the mother. Considering the abnormal biochemical indicators during pregnancy and CNV-related clinical phenotypes after birth, two male fetuses without prenatal diagnosis were suspected to carry the maternally-derived CNVs. Further, we identified three CNV-related family histories with variable phenotypes. Statistical analysis of the 61525 pregnancies revealed that Z-scores of chromosomes 21 and 18 were significantly associated with PPV at 3 ≤ Z ≤ 40. Notably, three pregnancies with Z > 40 were both maternal full aneuploidy. At Z < -3, fetuses carried microdeletions instead of monosomies. Sex chromosome trisomy was significantly higher PPV than monosomy.

**Conclusion:** The positive rate of the NIPT screening model combining Z-score and maternal CNV analysis increased from 6.91‰ (22/3184) to 12.25‰ (39/3184) and true positives increased from 12 to 21 pregnancies. We found that this method could improve the positive rate and accuracy of NIPT for aneuploidies and CNVs without increasing testing costs. It provides an early warning for the inheritance of pathogenic CNVs to the next generation.

## Introduction

Non-invasive prenatal testing (NIPT) based on high-throughput sequencing can detect fetal common chromosomal aneuploidies. The existence of placental cell-free fetal DNA (cff-DNA) fragments in the peripheral blood of pregnant women provide a basis for NIPT technology (Lo et al., 1997). Mostly, NIPT result was calculated by Z-score in which the individual sample is compared with a control group of normal (diploid) samples (Chiu et al., 2008). Numerous studies have shown that the model’s accuracy is higher than that of serological screening technology, regardless of single and twin pregnancies (Zhang et al., 2015; Gil et al., 2017; Iwarsson et al., 2017; Gil et al., 2019). However, there are false results that cannot be avoided owing to the limitations of the materials and methods. Several studies indicated the accuracy and positive predictive value (PPV) of NIPT were related to Z-score; and the higher the Z-score, the greater the likelihood of true positive (Tian et al., 2018; Wan et al., 2021). Another study showed that the optimal cut-off values for trisomy (T) 21 and T18 Z-scores were 5.79 and 6.05, respectively (Zhou et al., 2021), which PPV in the group of Z-score > optimal cutoff value was higher than that in the group of 3 ≤ Z-score < optimal cutoff value. However, increasing the cut-off value will produce more false negatives. Therefore, more research is necessary on the relationship between Z-score and PPV before adjusting the cut-off value.

Maternal copy number variations (CNVs) were ignored in either NIPT or NIPT-plus except identifying of fetal *de novo* CNVs. Pathogenic CNVs cause over 300 types of chromosomal microdeletion/microduplication syndromes (MMS), with a total incidence of 3% (Weise et al., 2012; Nevado et al., 2014; Levy et al., 2018). It is very common for heterogeneity of clinical feature due to the location and size of CNVs. A few of fetal structural abnormalities resulted from microdeletion/microduplication could be found by ultrasound screening, however, most of the MMS could not be identified during pregnancy (Grati et al., 2015). Recently, there were studies to shown that the PPV of fetal CNVs detected by NIPT-plus was 20–40% (Chen et al., 2019; Hu et al., 2019). However, no research pay attention to pathogenic CNVs inherited from parents. We found maternal CNV analysis through NIPT without additional cost was meaningful for prediction of birth defects and future treatment.

This prospective study explored a new NIPT model combining Z-score and maternal CNV analysis to identify high-risk fetuses, including aneuploidies and CNVs of each chromosome. In addition, we investigated whether the Z-score was correlated with PPV to assess the accuracy of NIPT-positive results from a single center within the past 5 years. This study aimed to provide a more accurate basis for clinical genetic counselling.

## Materials and Methods

### Subjects

Pregnant women selected for NIPT in Changzhi Maternal and Child Health Care Hospital were continuously included in this study from October 2016 to November 2021. Testing was successful in 61,525 pregnant women, of which 32,361 pregnancies from October 2016 to June 2019 were derived from (Li et al., in press). The study included pregnant women with aneuploidy to investigate the effects of maternal aneuploidy on the Z-score, and we also recommended that pregnant women participated simultaneously in invasive prenatal diagnosis. A total of 3,184 pregnant women were selected from August to November 2021 for NIPT combining Z-score and maternal CNV analysis (CNV > 0.5 Mb). All pregnant women voluntarily signed informed consent forms prior to the procedure. Unique identifiers were deleted before they were included in the study. All procedures were approved by the Medical Ethics Committee of Changzhi Maternal and Child Health Care Hospital (No. CZSFYLL2021017).

### Noninvasive Prenatal Testing

Plasma was separated via a two-step centrifugation process within 72 h after collecting 5–8 ml maternal peripheral blood using a dedicated cell-free DNA collection tube. After cf-DNA extraction, library construction, and pooling, samples were sequenced on the Illumina NextSeq CN500 or NextSeq 550Dx platforms in collaboration with Findgene (Shanghai, China) or Biosan (Hangzhou, China). Sequences were aligned to the human genome-wide standard sequence (GRCh37) using BWA software, and Z-scores for each chromosome were obtained from bioinformatics analysis. Z-score were corrected by a series of bioinformatics methods such as normalization, GC correction and filtering out maternal CNVs. But it cannot correct for maternal aneuploidy interference. The control used a non-fixed reference set (96 experimental samples per batch) for internal comparison to eliminate batch differences. Qualified samples required the row sequencing reads greater than 3.5Mb and the fetal frequency greater than 4%. The thresholds of aneuploidy were ±3. Below the lower limit indicated a high risk of monosomy, above the upper limit indicated a high risk of trisomy. Between -3 and +3 represented a low risk of aneuploidy. Interpreting results from sex chromosome aneuploidies (SCAs) required combining the Z-scores of chromosome (Chr) X and ChrY. The Z-scores of ChrX and ChrY should be between −3 and 3 in normal female fetuses. The Z-scores of ChrX and ChrY in normal male fetuses should be < −3 and >3, respectively. All cases with positive results for the first time were verified in another plasma, and only the verified results were included in statistical analyses.

### Analysis of Maternal Copy Number Variations

CNVs were detected using sliding window algorithm counting reads in each continuous bins (100kb size). To verify that the method for maternal CNVs is reliable, we performed genomic testing of maternal own lymphocytes by CNV-seq or SNP-array technology. CNVs were classified according to the technical standard for the interpretation and reporting of constitutional copy-number variants by the American College of Medical Genetics and Genomics (ACMG) and the Clinical Genome Resource (ClinGen) version 2020 (Riggs et al., 2020). Maternal phenotype and family history were assessed through genetic counselling. Prenatal diagnosis was recommended for pregnancies with maternal CNVs.

### Prenatal Diagnosis

Prenatal diagnosis was recommended in pregnant women with NIPT-positive. Amniocentesis was performed under ultrasound guidance at 18–23 gestational weeks or umbilical blood was performed when over 23 gestational weeks, with the consent of pregnant women and family members. Prenatal diagnosis techniques were chosen by one or a combination of karyotyping, SNP-array or CNV-seq. Operations and analyses were performed in accordance with relevant international and national guidelines. The detailed precedures were applied as previously reported (Ma et al., 2021).

### Follow-Up

Pregnant women with NIPT-positive results who were not prenatally diagnosed at our institution were followed up to confirm the prenatal diagnosis at other institution or to perform postnatal diagnosis if they chose to continue their pregnancies.

### Statistics

Logistic regression analysis was applied to associate Z-scores with the PPV of NIPT-positive results. The receiver operating characteristic (ROC) curve is a comprehensive index that reveals the relationship between sensitivity and specificity. The larger the area under the curve (AUC), the higher the accuracy. Analyses were in the R version 4.1.2. The trend of S-curves was drawn based on the Z-scores and PPV in MATLAB version R2021b with a single parameter logistic model, using the function $f\left(x\right)=\frac{1}{1+a_{0}e^{-x}}$ .

## Results

### Efficiency of the New Model Combining Z-Score and Maternal CNV

We combined Z-score and maternal CNV to analyze data from 3,184 pregnancies with 3,090 singletons and 94 twins, and 90 pregnancies using assisted reproductive technology. Participants were 30.24 ± 4.46 years old (range, 16–46 years). As shown in Table 1, a total of 22 pregnancies (all singletons) were positive, with outlier Z-scores (Z < -3 or Z > 3) suggesting fetal aneuploidy. Twelve were true positives (PPV = 54.5%), including seven with T21, one with T18, one with T13, two with SCAs, and one with T9. Maternal CNV analysis showed that 17 pregnant women had pathogenic or likely pathogenic CNVs, interpreted through websites such as DECIPHER (https://www.deciphergenomics.org) and ClinGen (https://dosage.clinicalgenome.org) in Table 2. They did not overlap with the 22 positives analyzed using Z-scores. The NIPT-positives increased from 22 (6.91‰) analyzed using Z-scores to 39 (12.25‰) analyzed using Z-scores and maternal CNVs. The consequences of validation by maternal own lymphocytes were consistent (Supplementary Table S1). Among 17 maternal CNVs group, 10 pregnant women chose prenatal diagnosis, of that seven fetuses carried the same CNVs as their mother, indicating that 70% (7/10) CNVs were passed to the next generation. The other seven pregnant women refused prenatal diagnosis. Of two male fetuses undiagnosed exhibited extremely low levels of unconjugated estriol (uE3) during pregnancy, a biochemical indicator of the X-linked ichthyosis (caused by the CNVs), and present skin symptoms after birth; thus we suspected them of having the same CNVs as their mothers. Through genetic counseling, we found that some pregnant women exhibited CNV-related phenotypes in themselves or relatives what they ignored before.

**TABLE 1:** Results from the Cohort of 3184 Pregnancies with NIPT Combining Z-score and Maternal CNV.

Groups	Number of Outlier Z-scores						Number of maternal CNVs				Total (PR)	
	T21	T18	T13	SCAs	OAAs	Total (PR)		Del	Dup	Total (PR)			
NIPT+	7	4	1	6	4	22 (6.91‰)		12	5	17 (5.34‰)		39 (12.25‰)	
TP	7	1	1	2	1	12		5^*^	4	9^*^		19	

^*^Including 2 fetuses with phenotypes related-CNV without genomic diagnosis.

NIPT, Non-invasive Prenatal Testing; NIPT+, NIPT positive result; CNVs, copy number variations; T, trisomy; SCAs, Sex chromosome aneuploidies; OAAs, Other autosome aneuploidies (excepting Chr21, Chr18 and Chr13); PR, positive rate; Del, Microdeletion; Dup, Microduplication; TP, true positive.

**TABLE 2:** 17 Maternal CNVs Detected by NIPT and Fetal Diagnosis Results.

Sample ID	Maternal CNV	size (Mb)	Z_bc_	Z_ac_	CNV Interpretation	Maternal Phenotypes	Fetal Diagnosis
21J101249	seq[GRCh37]chr22:g.21706150-24644732 x1	2.94	−5.193	0.548	Contained 92 protein-coding genes, a 3-point HI gene and a 3-point HI genomic, a variable clinical phenotype such as global developmental delay, cleft lip, behavioral problems and mild dysmorphic facial features.	Slight facial asymmetry, communication impairment, her daughter suffered from cleft palate and mental/physical retardation.	seq[GRCh37]chr22:g.21702383-24620002x1
21J104405	seq[GRCh37]chr16:g.14889818-16535522 x3	1.65	1.414	0.227	Contained 16 protein-coding genes, a 2-point TS genomic region, a variable clinical presentation, lower penetrance.	No obvious abnormality.	arr[GRCh37]chr16:g.15406415-16282869x3
21J101817	seq[GRCh37]chr4:g.182695733-189079179 x1	6.38	−6.731	−0.428	Contained 37 protein-coding genes, symptomatic seizures, short stature.	Height less than 150cm, No obvious abnormality in intelligence.	arr[GRCh37]46,XN
21J104345	seq[GRCh37]chr17:g.14161233-15458439 x1	1.30	−2.137	−0.529	Contained 6 protein-coding genes, a 3-point HI gene and a 3-point HI genomic region, hereditary neuropathy with liability to pressure palsies (HNPP), few symptoms on many individuals.	No obvious abnormality; her deceased mother suffered from leg discomfort after middle age.	arr[GRCh37]chr17:g.14099565-15482833x1
21J101676	seq[GRCh37]chrX:g.2795214-17648380 x1	14.85	−7.708	0.884	Contained 66 protein-coding genes, 9 3-point HI genes and a 3-point HI genomic region, female carriers were unaffected or milder phenotypes.	Abortion history, This pregnancy is a female fetus.	seq[GRCh37]46,XN
21J105324	seq[GRCh37]chrX:g.6445119-8104085 x1	1.69	−17.549	−17.768	Contained 5 protein-coding genes, a 3-point HI genomic region, X-linked ichthyosis in males, mild or unaffected in females.	No obvious abnormality. This pregnancy was a male fetus and MoM value of uE3 was 0.08 (Standard Range, >0.7).	No prenatal diagnosis; Skin lesions on limbs 2 months after birth.
21J104580	seq[GRCh37]chr2:g.111195659-113121587 x3	1.93	−0.205	−1.512	Contained 11 protein-coding genes, a 2-point TS genomic region, a variable clinical phenotypes including developmental delay, tooth abnormalities, hypotonia, and neuropsychiatric conditions.	No obvious abnormality except tooth abnormality	No prenatal diagnosis
21J104606	seq[GRCh37]chr16:g.29410978-30305956 x3	0.89	2.325	1.792	Contained 37 protein-coding genes, a 3-point TS genomic region, a variable clinical presentation, incomplete penetrance.	No obvious abnormality	arr[GRCh37]chr16:g.29589674-30176508x3
21J107686	seq[GRCh37]chrX:g.6472218-8150233 x1	1.68	-6.918	−10.015	Contained 5 protein-coding genes, a 3-point HI gene and a 3-point HI genomic region, X-linked ichthyosis in males, mild or unaffected in females.	No obvious abnormality. This pregnancy was a male fetus and MoM value of uE3 was 0.03 (Standard Range, >0.7).	No prenatal diagnosis; Dry and rough skin 10 days after birth.
21J108961	seq[GRCh37]chr17:g.34710859-36306985 x1	1.60	−3.974	-1.550	Contained 18 protein-coding genes, contained a 3-point HI genomic region, renal cysts and diabetes syndrome, incomplete penetrance.	Renal cyst, congenital abnormal splenic structure and gallstones	No prenatal diagnosis
21J108971	seq[GRCh37]chr15:g.23990956-28419527 x3	4.43	5.978	−0.757	Contained 10 protein-coding genes, overlapped a 3-point HI genomic region, intellectual disability, psychiatric disorders, phenotypes by maternally-derived.	Mild schizophrenia, her brother had obvious mental retardation.	arr[GRCh37]chr15:g.23632678-28526905x3
21J104969	seq[GRCh37]chrX:g.2795214-16240667 x1	13.45	−9.986	−0.384	Contained 59 protein-coding genes, contained 9 3-point HI genes and a 3-point HI genomic region, Female carriers were unaffected or milder phenotypes.	Abortion history, this pregnancy is a female fetus.	No prenatal diagnosis
21J107005	seq[GRCh37]chr16:g.15112139-16561127 x1	1.45	−0.934	0.195	Contained 14 protein-coding genes, a 3-point HI genomic region, phenotypic variability, incomplete penetrance.	No obvious abnormality	No prenatal diagnosis
21J100568	seq[GRCh37]chr16:g.15142813-16428637 x1	1.29	−1.161	0.019	Contained 13 protein-coding genes, a 3-point HI genomic region, phenotypic variability, incomplete penetrance.	No obvious abnormality, her son was intellectual and language disability.	arr[GRCh37]chr16:g.15481748-16458424x1
21J105304	seq[GRCh37]chr17:g.14126371-15556920 x3	1.43	2.386	0.170	Contained 10 protein-coding genes, a 3-point HI genomic region, Charcot-Marie-Tooth syndrome (CMT) characterized by slowly progressive.	No obvious abnormality	arr[GRCh37]chr17:g.14087918-15428901x3
21J104462	seq[GRCh37]chr2:g.111476219-113095275 x1	1.62	−3.545	−2.115	Contained 8 protein-coding genes, overlapped a 2-point HI genomic region, clinical findings are variable, non-specific dysmorphic features.	Lost to follow-up	No prenatal diagnosis
21J106665	seq[GRCh37]chr16:g.15395056-18200933 x1	2.81	−3.178	−1.059	Contained 12 protein-coding genes, a 3-point HI genomic region, phenotypic variability ,incomplete penetrance.	No obvious abnormality	arr[GRCh37]46,XN

Z_bc_, Z-score before correction of the chromosome where the CNV is located; Z_ac_, Z-score after correction; uE3, unconjugated estriol; MoM, multiple of median.

We compared 17 maternal CNVs with their Z-scores to explore the effect of CNV on Z-score. Z-scores with maternal CNVs were different between before and after correction. Z-scores before correction shown that 6 out of 13 autosomes and 2 out of 4 chromosome X had outlier Z-scores. Of maternal CNVs below 2 Mb size, 9 out of 11 had Z-scores before correction in the normal range. Of maternal CNVs above 2Mb, all of 6 were outliers with Z-scores before correction. It is noteworthy that Z-scores after correction were all negative. These CNVs would be missed if only concerned Z-scores.

### General Analysis of NIPT-Positive Results Using Z-Scores

Z-score analysis without or maternal CNV analysis, was applied to the cohort of 61,525 pregnancies. Maternal and fetal characteristics, along with positive results, are shown in Table 3. We found outlier Z-scores in 402 pregnancies, and the positive rate was 6.53‰. Abnormalities of Chr21, Chr18, Chr13, SCAs, and other autosomes accounted for 61.03% (214/402), 16.92% (68/402), 7.21% (29/402), 14.18% (57/402), and 8.46% (34/402), respectively. Of the 402 pregnancies, 303 underwent prenatal or postnatal diagnoses, 44 pregnancies were miscarrige or induced labour due to structural abnormalities, and 55 pregnancies were lost to follow-up. PPV for twin pregnancies did not significantly differ from singletons, with 83.33% (5/6) of twin and 70.37% (209/297) of singleton. Overall PPV was 70.20%, of which Chr21, Chr18, sex chromosomes, Chr13, and other autosomes were lower successively with 86.21% (150/174), 66.67% (32/48), 50.00% (20/40), 33.33% (7/21), 25.00% (5/20), respectively.

**TABLE 3:** Characteristics and Results from the Cohort of 61525 Pregnancies

	Cohort	NIPT+	Diag	TP
Maternal and fetal characteristics				
Age (year)	29.94 ± 4.86	30.91 ± 5.67	30.67 ± 5.44	30.88 ± 5.46
GW (week)	18.97 ± 2.23	18.77 ± 2.32	18.83 ± 2.17	18.56 ± 2.18
ART (%)	1230 (2.00)	4 (1.00)	4 (1.32)	3 (1.40)
Twin (%)	1763 (2.87)	9 (2.24)	6 (1.98)	5 (2.34)
Number of Outlier Z-scores				
Total	−	402	303	214
Chr21	−	214	174	150
Chr18	−	68	48	32
Chr13	−	29	21	7
SCAs	−	57	40	20
OAAs	−	34	20	5

Diag, Fetuses with diagnostic testing; GW, Gestational week; ART, assisted reproductive technology; Chr, chromosome.

We found outlier Z-score did not always indicate fetal aneuploidy. In this study, six pregnancies with outlier Z-scores were fetal CNVs verified by prenatal diagnosis, four were mosaic aneuploidies and three false positives were caused by maternal aneuploidies. An extreme outlier Z-scores for ChrY (Z = 362) were discovered in a pregnant woman with a history of bone marrow transplantation.

### Accuracy Analysis of Z-Scores for Chr21, Chr18, and Chr13

**FIGURE 1:**
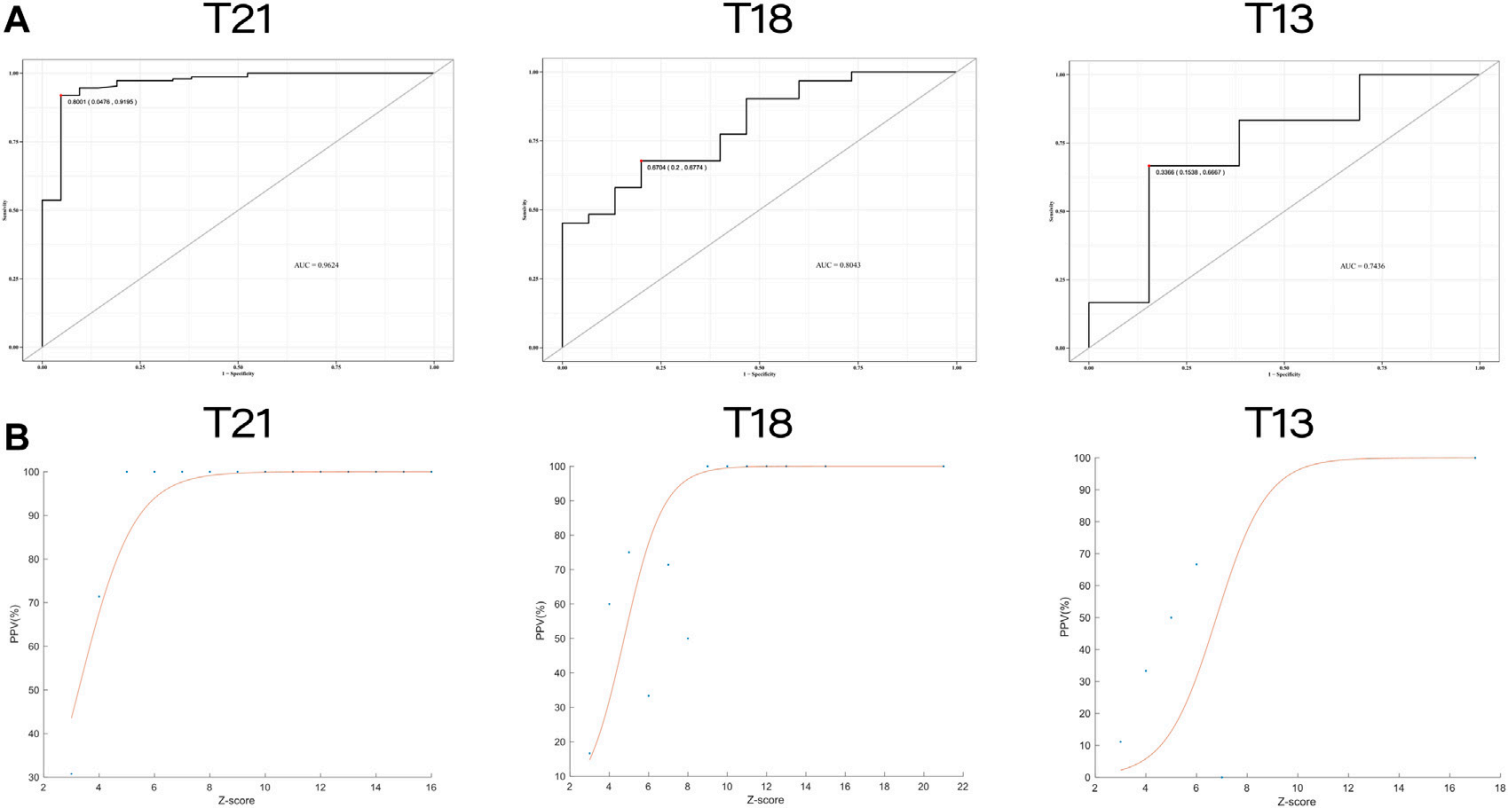
The receiver operating characteristic (ROC) curves and trend curves between Z-score and the positive predictive value. **(A)** The ROC curves of T21, T18, and T13. The area under the curve (AUC) were 0.9624, 0.8043 and 0.7436, respectively. **(B)** The trend curves of T21, T18 and T13 with S-curve model ( $f\left(x\right)=\frac{1}{1+a_{0}e^{-x}}$ ).

The Chr21, Chr18, and Chr13 positive pregnancies were divided into six groups according to Z-scores: Z ≤ -3, 3 ≤ Z ≤ 4, 4 < Z ≤ 5, 5 < Z ≤ 6, 6 < Z ≤ 40, and Z > 40 (Table 4 **)**. At Z-scores of 3 ≤ Z ≤ 40, PPV increased with increasing Z-scores. At the same Z-score, the PPV of Chr21 was always the highest and that of Chr13, was lowest. Notably, two pregnancies with Z > 40 were both maternal Down syndrome. With Z-scores < -3, fetuses carried microdeletions instead of monosomy. As shown in Table 5, logistic regression analysis revealed that Z-scores were significantly associated with the PPV of T21 (OR = 3.752, *p* < 0.001) and T18 (OR = 1.532, *p* = 0.00817). In the ROC curves, AUCs of T21, T18, and T13 were 0.9624, 0.8043, and 0.7436, respectively. S-curves were simulated to predict the trend of PPV shown in Figure 1. We also revealed several special types of karyotype by diagnosis, including Robertsonian translocation, mosaic trisomy, and CNVs; these cases exhibited outlier Z-scores in NIPT.

**TABLE 4:** Comparison of NIPT Results and Diagnoses in Different Groups.

Chr	Group	N	Diagnosis result and Number		PPV (%)
21	Z ≤ -3	2	No abnormality	2	0
	3 ≤ Z ≤ 4	26	T21	8	30.77
			No abnormality on Chr21, but 2.2Mb deletion on 9p12	1	
			No abnormality on Chr21, but 7.7Mb duplication on Xp21.3-p21.1	1	
			No abnormality, including 1 twin	15	
			No abnormality with postpartum follow-up	1	
	4 < Z ≤ 5	7	T21	4	71.43
			Approximately 6Mb duplication on 21q21.1	1	
			No abnormality	2	
	5 < Z ≤ 6	11	T21	9	100.00
			Mosaic T21 and mosaic X0	1	
			T21 and 1.4Mb deletions on 17p12	1	
	6 < Z ≤ 40	126	T21	117	100.00
			T21 on one of twin	4	
			T21 with rob (21;21)	1	
			Mosaic T21	1	
			Approximately 10Mb duplication on 21q11.2-q21.2	1	
			T21 in postpartum follow-up	2	
	Z > 30	2	No abnormality, but T21 on mother	2	0
18	Z ≤ -3	2	6.0Mb deletion on 18p11.32-p11.31	1	50.00
			No abnormality	1	
	3 ≤ Z ≤ 4	6	T18	1	16.67
			No abnormality	5	
	4 < Z ≤ 5	10	T18	6	60.00
			No abnormality	4	
	5 < Z ≤ 6	4	T18	3	75.00
			No abnormality on Chr18, but 33.3Mb duplication on Chr11	1	
	6 < Z ≤ 40	26	T18	19	80.77
			Mosaic T18	1	
			3.1Mb duplication on 18p11.32-p11.31	1	
			No abnormality	5	
13	Z ≤ -3	2	10.6Mb deletion on 13q21.1-q21.32	1	50.00
			No abnormality on Chr13, but 0.9Mb duplication on Chr16	1	
	3 ≤ Z ≤ 4	9	1.5Mb duplication on 13q12.12	1	11.11
			No abnormality	7	
			No abnormality in postpartum follow-up	1	
	4 < Z ≤ 5	3	T13	1	33.33
			No abnormality	2	
	5 < Z ≤ 6	2	T13	1	50.00
			No abnormality	1	
	6 < Z ≤ 40	5	T13	3	60.00
			No abnormality on Chr13, but 1.4Mb duplication on 7p21.3	1	
			No abnormality	1	

Chr, chromosome; N, number; PPV, positive predictive value.

**TABLE 5:** Correlation between Z-score and Positive Predictive Value (PPV) by Logistic Regression Analysis.

Group	B	Wald	OR	95% CI	*P*
T13	0.465	1.742	1.592	1.001–3.551	0.1869
T18	0.427	6.996	1.532	1.174–2.229	0.00817
T21	1.322	18.73	3.752	2.282–7.811	<0.001

B, beta coefficients; Wald, wald test; OR, odds ratio; Cl, confidence interval; P, p-value; T, trisomy.

### Accuracy Analysis of Z-Scores for Sex Chromosomes and Other Autosomes

In 40 pregnancies with abnormal Z-scores for sex chromosomes, the PPV was 50.00% (20/40) shown in Table 6. Among them, the PPV of sex chromosome trisomy was 64.00% (16/25), and that of monosomy was 26.67% (4/15), with a significant difference (*p* < 0.05). Five out of 20 for other autosomal abnormalities were true positive (PPV = 25%) diagnosed by SNP-array or CNV-seq technology, including aneuploidies (T16 and mosaic T16) and CNVs on Chr9 and Chr10.

**TABLE 6:** Comparison of NIPT Results and Diagnoses in Sex Chromosomes and Other Autosomes.

Chr	Type	N	Diagnostic result and Number		PPV (%)
SCAs	X0	15	X0	1	26.67%
			X0/XN	2	
			X0/X,r(X)	1	
			No abnormality	11	
	XXX	16	XXX	9	56.25
			No abnormality, but XXX is found on mother	1	
			No abnormality	6	
	XYY	1	XYY	1	100.00
	XXY	8	XXY, including 1 twin-sample	6	75.00
			No abnormality	2	
	**Total**	**40**		**20**	**50.00**
OAAs	T2	1	No abnormality	1	
	T3	1	No abnormality	1	
	T7	5	No abnormality	5	
	T8	3	No abnormality	3	
	M9	1	20Mb deletion on 9p24.3p21.3 and 37Mb deletion on 9q21.13q31.3	1	
	T9	1	38.6Mb duplication on 9p24.3p13.1	1	
	T10	1	0.3Mb duplication on 10q23.33	1	
	M14	1	No abnormality	1	
	T15	1	No abnormality	1	
	T16	2	T16	1	
			Mosaic T16	1	
	M16&19	1	No abnormality	1	
	T20	1	No abnormality	1	
	M22	1	No abnormality	1	
	**Total**	**20**		**5**	**25.00**

M, monosomy.

## Discussion

Because PPV indicates the possibility of true positive, it is usually used to evaluate the predictive ability of the test (Meck, et al., 2015; DiNonno, et al., 2019). PPV affected by factors such as population size and region, given that it is related to a population’s basic prevalence (Monaghan, et al., 2021).

Previous studies using the ion proton semiconductor platform and the BGISEQ-500 sequencing platform suggested that Z ≥ 9/10 had a higher PPV(Tian et al., 2018; Wan et al., 2021). However, there are potential differences in low Z-scores between different sequencing platforms. In this study, we performed Z-score grouping, logistic regression analysis, ROC curves and S-curve trends to determine correlations between Z-score and PPV. There was the significant correlations between Z-scores and PPV at T21 and T18, with the exception of T13. In addition, we found that the true positives in Z < −3 were all microdeletions instead of monosomy, which was not mentioned before. Because of the diversity in sex chromosomal and other autosomal abnormalities, more data are necessary to increase the accuracy of observed trends.

**FIGURE 2:**
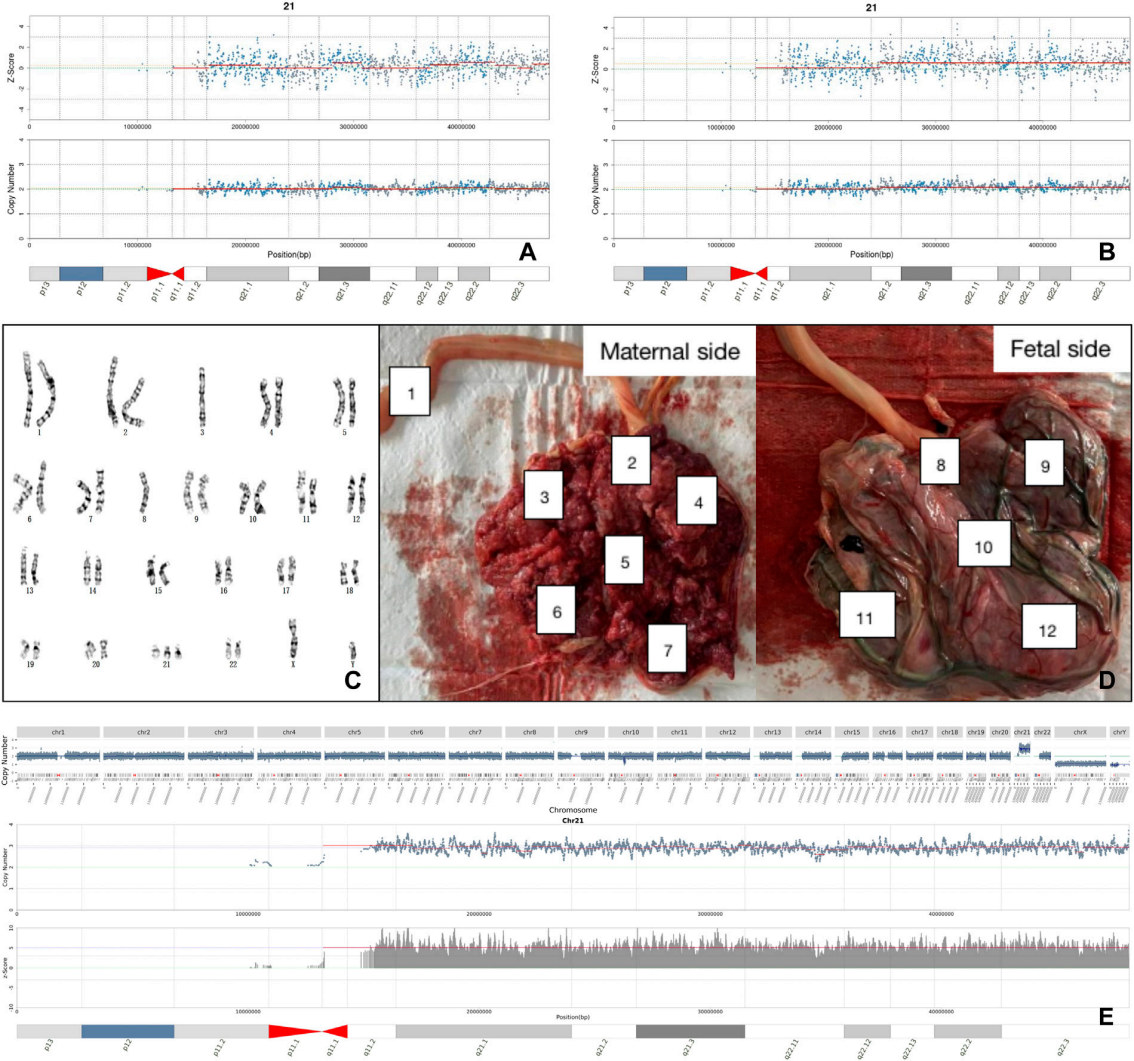
Analysis of a false negative case. **(A)** The negative result of NIPT with first blood sampling. **(B)** The positive result of NIPT with re-sampling. **(C)** The karyotype of fetal amniotic fluid cell. **(D)** Sampling locations of placental tissue after labor induction. **(E)** Placental CNV-seq results suggested full T21.

Several factors affect the accuracy of NIPT. Confined placental mosaicism (CPM), with an incidence of 1–2%, is a common reason (Malvestiti et al., 2015; Mardy et al., 2016). Another reason is maternal chromosomal abnormalities (Wang et al., 2014; Zhou et al., 2017). A study found that maternal CNVs could increase the false positive rate of NIPT by 10% (Snyder et al., 2015). Our study found three pregnancies with Z-scores > 40, were all false positive caused by maternal full aneuploidy. A common cause of false negatives is the low fetal frequency, where the cff-DNA increases as gestational weeks increase. Following previous research, we recollected blood samples in cases of inadequate fetal frequency (Wang et al., 2013). A false-negative case was found in our study due to low fetal frequency (FF=3.8%, Z < 3). Unexpectedly, the fetal frequency reached 9.6% re-collected after two weeks and Z-score of chromosome 21 was 6.66 which indicated that the fetus was T21 positive. The fetus was verified by prenatal diagnosis as 47,XN,+21. For this case, we also used CNV-seq on placental tissue to yield a results of 47,XY,+21, thus excluding CPM shown in Figure 2. Another factor that can interfere with NIPT results is history of transplantation (Zhu et al., 2021). In our study, a pregnant woman received a bone marrow transplant from a male donor 7 years ago. The Z-score of ChrY was 362, far exceeding Z-score from typical male fetuses (Z-score = 3–150). Prenatal diagnosis indicated a nomal fetal karyotype. We further explored cell sources of this pregnant woman’s peripheral blood, oral cavity, and hair follicle cells using STR markers in sex chromosomes, and found different proportions of cell sources. The hair follicle cells were all from the pregnant woman herself, the peripheral blood lymphocytes were all from the bone marrow donor, and the oral cells were the co-existence of the two sources.

Maternal CNVs were seldom researched in NIPT or NIPT-plus technology. NIPT-plus can identify fetal de novo MMS with 0.174% positive rate (Liang et al., 2019). Considering the incidence of pathological CNVs (1.7%) in all pregnancies with normal fetal structures (Wapner et al., 2012), the inherited CNVs were underestimated. By maternal CNV analysis in this study, we found that maternally-inherited CNVs with clinically significant reached 0.28% (9/3184) comprising 7 fetuses with prenatal diagnosis and 2 with specific phenotypes after birth. It was valuable of increasing detection rate of NIPT without increasing testing costs.

**FIGURE 3:**
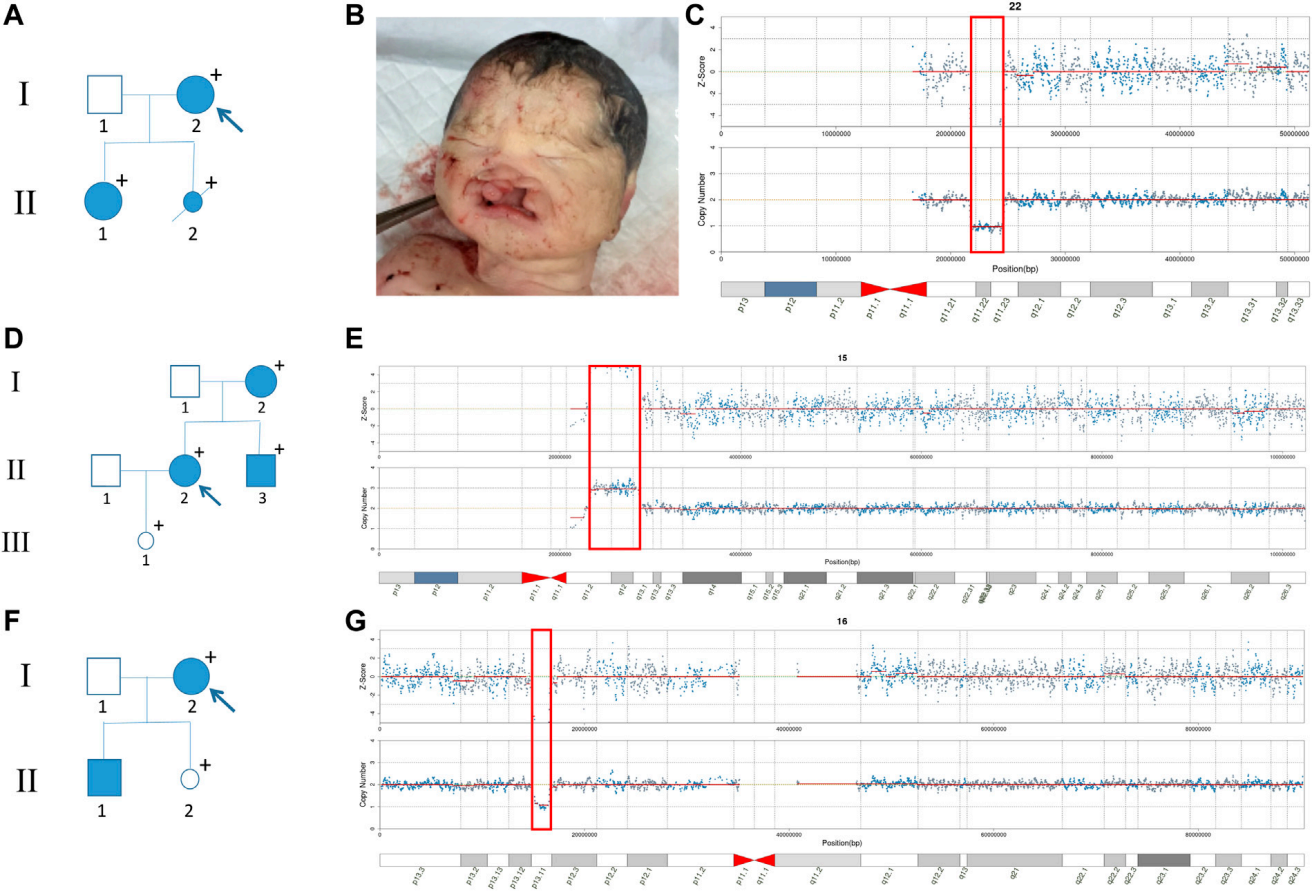
Family pedigrees with maternal CNVs. **(A)** Family pedigrees of No. 21J101249. **(B)** The facial phenotype of post-induction fetus (No. 21J101249), including cleft lip and palate. **(C)** Maternal CNV of No. 21J101249 suggested by NIPT. **(D)** Family pedigrees of No. 21J108971. **(E)** Maternal CNV of No. 21J108971 suggested by NIPT. **(F)** Family pedigrees of No. 21J100568. **(G)** Maternal CNV of No. 21J100568 suggested by NIPT.

Although some pregnant women did not show obvious symptoms due to incomplete penetrance of CNVs, further genetic counseling may reveal mild phenotypes or severe family histories. In our study, the pregnant woman of No. 21J101249 (Figure 3A, I-2) had slight facial asymmetry and communication impairment. Her daughter (II-1) suffered from congenital incomplete cleft palate, as well as mental and physical retardation with a low developmental quotient (DQ = 62). The fetus was induced due to severe cleft lip and palate (Figure 3B). Among I-2, II-1, and II-2, we found an approximately 2.9 Mb heterozygous deletion in 22q11.21-q11.23 (Figure 3C). SMARCB1 gene and 22q11.2 recurrent region (distal type I,D-E/F) in the fragment have sufficient evidence for haploinsufficiency, which are associated with clinical phenotypes such as global developmental delay, intellectual disability, cleft lip, and behavioral problems (Mikhail et al., 2014; Holsten et al., 2018). The pregnant woman of No. 21J108971 (Figure 3D, II-2) had mild schizophrenia; her brother (II-3) exhibited obvious mental retardation, but her mother (I-2) had no obvious abnormality. SNP-array technology revealed that I-2, II-2, and II-3 harbored an approximately 4.4 Mb heterozygous duplication in 15q11.2-q13.1 (Figure 3E), and analysis of fetal amniotic fluid cells (III-1) indicated that the fetus carried the same CNV 15q11.2q13 recurrent (PWS/AS) region (Class 1, BP1-BP3) and 15q11.2q13 recurrent (PWS/AS) region (Class 2,BP2-BP3) in the fragment have sufficient evidence for triplosensitivity, associated with autism, intellectual disability, seizures, and psychiatric disorders (Christian et al., 2008; Ingason et al., 2011). Evidence suggests a parent-of-origin effect, with maternally-derived duplications being more frequently associated with abnormal phenotypes. While the pregnant woman of No. 21J100568 (Figure 3H, I-2) had no obvious abnormality, prenatal diagnosis indicated that the fetus (II-2) carried the same CNV on 16p13.11 (Figure 3G). Additionally her first son suffered from retarded intellectual and language development that had not been detected (II-1) 16p13.11 recurrent region (BP2-BP3) (includes MYH11) in the fragment has sufficient evidence for haploinsufficiency, associated with intellectual disability and/or multiple congenital anomalies (de Kovel et al., 2010; Jähn et al., 2013). It showed sex-limited effect on the penetrance with a significant enrichment among male cases (Tropeano et al., 2013). The fetus had been born for one month without abnormality. The pregnant woman of No. 21J108961 with microdeletion on 17q12 had a renal cyst, congenital abnormal splenic structure, and gallstones but continued her pregnancy without a prenatal diagnosis. The pregnant woman of No. 21J101817 with microdeletion on 4q34.3-q35.2 are less than 150 cm tall despite no obvious developmental delay. The pregnant women of No. 21J101676 and No. 21J104969 had deletions on ChrX (CNVs >10 Mb) which can cause male lethality but no effect on female carriers. The pregnant woman of No.21J105324 and No.21J107686 had microdeletions on Xp22.31 and were pregnant with male fetuses. Inheritance of these CNVs by the male fetuses could result in X-linked ichthyosis caused by haploinsufficiency of STS gene. Additionally, we found that their uE3 was far below normal. studies have shown that a characteristic of ichthyosis is significantly reduced uE3 during the embryonic period (Kashork et al., 2002).

**FIGURE 4:**
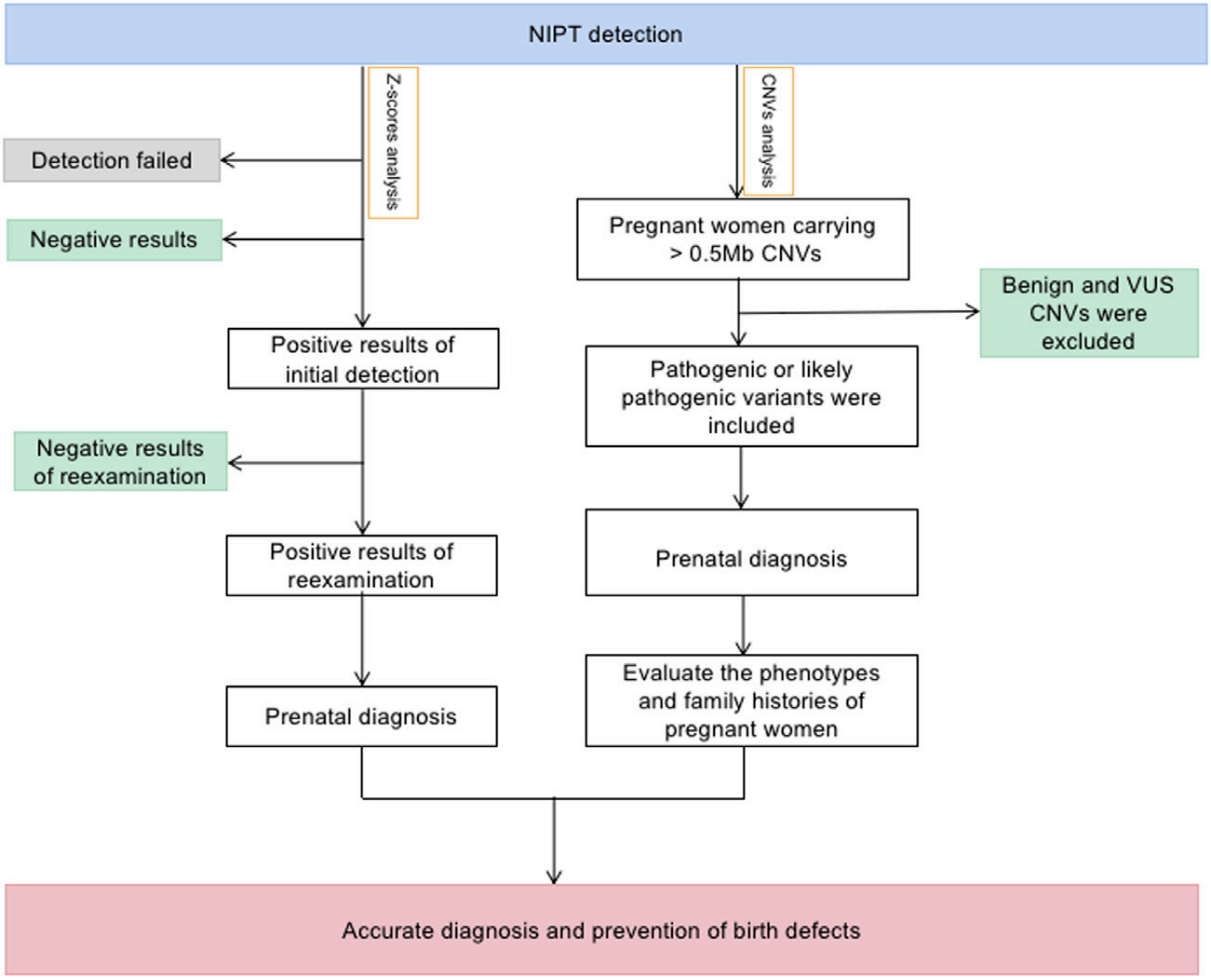
The flow of NIPT combining Z-score and maternal CNVs analysis.

The screening act as an early warning for parents regarding pathogenic CNVs that may be passed down to their offspring. Despite the small number of samples, our combined analysis increased NIPT positive rates from, 6.91‰ to 12.25‰ and found 9 clinically significant fetal CNVs which inherited from mothers without increasing detection costs and placing more economic pressure on pregnant women. Therefore, we propose a new NIPT screening model that integrates Z-scores and maternal CNVs (Figure 4).

## Data Availability

The datasets presented in this study can be found in online repositories. The names of the repository/repositories and accession number(s) can be found below: NCBI SRA BioProject, accession No:PRJNA837410.
