# Supplementary material for: Combining Z-Score and Maternal Copy Number Variation Analysis Increases the Positive Rate and Accuracy in Non-Invasive Prenatal Testing
Source: Front Genet. 2022 Jun 2;13:887176. doi: 10.3389/fgene.2022.887176 (PMC9201951; doi:10.3389/fgene.2022.887176)
Supplement: Supplementary file 2 [file Table1.pdf]

Supplementary Table S1. The consequences of validation by maternal own lymphocytes

No	Sample ID	Maternal CNV by NIPT	CNV-seq Calling by Maternal Peripheral Blood Lymphocytes
1	21J101249	seq[GRCh37]chr22:g.21706150-24644732 x1	seq[GRCh37] dup(22)(q11.21q11.23)chr22:g.21693572-24642475 x1
2	21J104405	seq[GRCh37]chr16:g.14889818-16535522 x3	seq[GRCh37] dup(16)(p13.11p13.11)chr16:g.14875122-16569327 x3
3	21J101817	seq[GRCh37]chr4:g.182695733-189079179 x1	seq[GRCh37] del(4)(q34.3q35.2)chr4:g.182654584-189106320 x1
4	21J104345	seq[GRCh37]chr17:g.14161233-15458439 x1	seq[GRCh37] del(17)(p12p12)chr17:g.14085269-15498451 x1
5	21J101676	seq[GRCh37]chrX:g.2795214-17648380 x1	seq[GRCh37] del(1)(p36.33p34.1)chr1:g.449163-44270605 x1 mos<br>seq[GRCh37] del(9)(q34.11q34.3)chr9:g.132569021-139454281 x1 mos<br>seq[GRCh37] del(X)(p22.33p22.13)chrX:g.2759596-17690771 x1<br>seq[GRCh37] del(X)(p22.13q28)chrX:g.17690771-155270560x1 mos
6	21J105324	seq[GRCh37]chrX:g.6445119-8104085 x1	seq[GRCh37] del(X)(p22.31p22.31)chrX:g.6437461-8121073 x1
7	21J104580	seq[GRCh37]chr2:g.111195659-113121587 x3	seq[GRCh37]dup(2)(q13q13)chr2:g.111210878-113151509 x3
8	21J104606	seq[GRCh37]chr16:g.29410978-30305956 x3	seq[GRCh37]dup(16)(p11.2p11.2)chr16:g.29369648-30284728 x3
9	21J107686	seq[GRCh37]chrX:g.6472218-8150233 x1	seq[GRCh37] del(X)(p22.31p22.31)chrX:g.6457925-8127011 x1
10	21J108961	seq[GRCh37]chr17:g.34710859-36306985 x1	seq[GRCh37] del(17)(q12q12)chr17:g.34640782-36195164 x1
11	21J108971	seq[GRCh37]chr15:g.23990956-28419527 x3	arr[GRCh38] 15q11.2q13.1(23387531-28281759) x3

12	21J104969	seq[GRCh37]chrX:g.2795214-16240667 x1	seq[GRCh37] del(X)(p22.33p22.2)chrX:g.2759596-16277969 x1
13	21J107005	seq[GRCh37]chr16:g.15112139-16561127 x1	No blood sample
14	21J100568	seq[GRCh37]chr16:g.15142813-16428637 x1	seq[GRCh37] del(16)(p13.11p13.11)chr16:g.14965376-16530613 x1
15	21J105304	seq[GRCh37]chr17:g.14126371-15556920 x3	seq[GRCh37] dup(17)(p12p12)chr17:g.14069455-15579516 x3
16	21J104462	seq[GRCh37]chr2:g.111476219-113095275 x1	seq[GRCh37] del(2)(q13q13)chr2:g.111187937-113127423 x1
17	21J106665	seq[GRCh37]chr16:g.15395056-18200933 x1	seq[GRCh37] del(16)(p13.11p12.3)chr16:g.15360618-18481647 x1
