# Supplementary material for: Combining Z-Score and Maternal Copy Number Variation Analysis Increases the Positive Rate and Accuracy in Non-Invasive Prenatal Testing
Source: Front Genet. 2022 Jun 2;13:887176. doi: 10.3389/fgene.2022.887176 (PMC9201951; doi:10.3389/fgene.2022.887176)
Supplement: Supplementary file 3 [file Table1.DOCX]

Supplementary Table1. Karyotype of the pregnant woman undergoing bone marrow transplantation

| Tissue Cells | Karyotype Results |
| --- | --- |
| Peripheral Blood Cells | 46,XY |
| Oral Epithelial Cells | 46,XX/46,XY |
| Hair Follicle Cells | 46,XX |
